# Impact of Vitamin D Deficit on the Rat Gut Microbiome

**DOI:** 10.3390/nu11112564

**Published:** 2019-10-24

**Authors:** Iñaki Robles-Vera, María Callejo, Ricardo Ramos, Juan Duarte, Francisco Perez-Vizcaino

**Affiliations:** 1 Department of Pharmacology, School of Pharmacy, Universidad de Granada, 18071 Granada, Spain; roblesverai@ugr.es (I.R.-V.); jmduarte@ugr.es (J.D.); 2 Ciber Enfermedades Cardiovasculares (CiberCV), 28029 Madrid, Spain; 3 Department of Pharmacology and Toxicology, School of Medicine, Universidad Complutense de Madrid, 28040 Madrid, Spain; maria.callejo@ucm.es; 4 Ciber Enfermedades Respiratorias (Ciberes), 28029 Madrid, Spain; 5 Instituto de Investigación Sanitaria Gregorio Marañón (IISGM), 28007 Madrid, Spain; 6 Fundación Parque Científico de Madrid, 28049 Madrid, Spain

**Keywords:** microbiota, 16S rRNA sequencing, vitamin D deficit

## Abstract

Inadequate immunologic, metabolic and cardiovascular homeostasis has been related to either an alteration of the gut microbiota or to vitamin D deficiency. We analyzed whether vitamin D deficiency alters rat gut microbiota. Male Wistar rats were fed a standard or a vitamin D-free diet for seven weeks. The microbiome composition was determined in fecal samples by 16S rRNA gene sequencing. The vitamin D-free diet produced mild changes on α- diversity but no effect on β-diversity in the global microbiome. Markers of gut dysbiosis like *Firmicutes*-to-*Bacteroidetes* ratio or the short chain fatty acid producing bacterial genera were not significantly affected by vitamin D deficiency. Notably, there was an increase in the relative abundance of the *Enterobacteriaceae*, with significant rises in its associated genera *Escherichia*, *Candidatus blochmannia* and *Enterobacter* in vitamin D deficient rats. *Prevotella* and *Actinomyces* were also increased and *Odoribacteraceae* and its genus *Butyricimonas* were decreased in rats with vitamin D-free diet. In conclusion, vitamin D deficit does not induce gut dysbiosis but produces some specific changes in bacterial taxa, which may play a pathophysiological role in the immunologic dysregulation associated with this hypovitaminosis.

## 1. Introduction

The gut microbiota is an ecological community constituted by a large number of bacteria, archaea, protists, fungi and viruses. The microbiota produces multiple metabolites that may cross the intestinal barrier and exert biological effects [1]. The microbiota also impacts on the integrity of the gut barrier. Gut barrier dysfunction may result in bacterial translocation from the intestines with increased plasma lipopolysaccharides (LPS) [2]. Multiple studies have found a relationship between the microbiota and adequate metabolic, hormonal and immunologic homeostasis [3,4,5]. Gut dysbiosis, i.e., an altered composition of the intestinal microbiota in disease is associated with poor health outcomes. Dysbiosis may be treated with probiotics, i.e., live strains of selected bacteria, or prebiotics, food components that modulate the microbiota [6,7]. Multiple metabolic, cardiovascular, and respiratory diseases including type 2 diabetes mellitus, obesity, systemic and pulmonary hypertension, atherosclerosis, heart failure and chronic respiratory diseases have been linked to impaired gut microbiota [8,9,10,11,12,13]. Dysbiosis is frequently characterized by: (a) lower microbial diversity and richness, (b) changes in the relative abundance of phyla with an increased *Firmicutes* to *Bacteroidetes* ratio (F/B), and (c) changes in short chain fatty acids (SCFA) producing bacteria with a relative increase in lactate-producing bacteria and a relative decrease in acetate- and butyrate-producing bacteria [2,4,14,15].

Vitamin D is produced from dermal exposure to sunlight or from the diet and is converted in the liver into 25-OH-cholecaciferol or calcifediol (25OHVitD), whose measure serves as an indicator of the vitamin D status. This is further metabolized mainly in the kidney into 1-α,25-dihydroxycholecalciferol, the most active form, also named calcitriol, which activates the vitamin D receptor (VDR). Vitamin D deficiency, which causes rickets and osteomalacia, is very common worldwide due to insufficient solar light exposure and/or a reduced dietary intake [16]. Besides this well-known regulatory role in the calcium-phosphorus and bone homeostasis, vitamin D is also involved in the control of other physiological processes, such as cellular growth, intracellular metabolism and innate and adaptive immunity. Vitamin D deficiency has also been related to infection, cancer and respiratory and cardiovascular diseases [17,18,19,20,21]. Immune cells and peripheral tissues can also synthetize calcitriol and express VDR. Thus, vitamin D has been proposed to have immunomodulatory properties and insufficient vitamin D levels may lead to dysregulation of immune responses [22]. Moreover, vitamin D is able to induce the expression of antibacterial proteins and exert antibiotic effects in a variety of cell types [23].

We hypothesized that a deficiency in vitamin D induces changes in the gut microbiota.

## 2. Materials and Methods 

The procedures involving animals were carried out according to the Spanish Royal Decree 1201/2005 and 53/2013 on the Care and Use of Laboratory Animals and approved by the institutional Ethical Committees of the Universidad Complutense de Madrid (Madrid, Spain) and the regional Committee for Laboratory Animals Welfare (Comunidad de Madrid, Ref. number PROEX-301/16). 

### 2.1. Model of Vitamin D Deficiency

Twenty male Wistar rats of 180 g body weight (BW) from Envigo (Barcelona, Spain) were maintained in the general animal facility of Universidad Complutense. Animals were randomly allocated into two groups; rats fed with a standard diet (*n* = 10), which contained 1500 IU/kg cholecalciferol (Teklad Global 18% Protein Rodent Diet, Envigo) and rats fed a vitamin D-free diet (*n* = 10) (VitD-free, Teklad Custom Diet TD.120008, Envigo, *n* = 10) for seven weeks. Rats were housed, two per box, with food and water ad libitum under standard conditions (22 ± 1 °C and 12:12 h dark/light cycle). At week 7, animals were euthanized, plasma and feces were collected and the right and left ventricle plus septum were weighed. Plasma 25OHVitD was measured using a chemiluminescence immunoassay (ADVIA Centaur^®^ Vitamin D Total assay, Siemens Healthcare Diagnostics) at the Clinical Biochemistry Service, Gregorio Marañón Hospital.

### 2.2. DNA Extraction, 16S rRNA Gene Amplification, Bioinformatics

The gut microbiome was analyzed as previously reported [13]. Briefly, the feces were collected at week 7 from each individual rat. We used G-spin columns (INTRON Biotechnology) to extract DNA and treated the samples with proteinase K and RNAses. The V3–V4 region of the 16S rRNA gene was amplified for 20–22 cycles from 3 ng of DNA [24]. PCR products (approx. 450 bp) included extension tails, which allowed sample barcoding and the addition of specific Illumina sequences in a second PCR for 10–12 cycles. Individual amplicon libraries were analyzed using a Bioanalyzer 2100 (Agilent) and a pool of samples was made in equimolar amounts. DNA samples were sequenced at the Unidad de Genómica (Parque Científico de Madrid) on an Illumina MiSeq instrument with 2 × 300 paired-end read sequencing. DNA reads were quality filtered according to MiSeq standard parameters (Illumina). We discarded all reads whose corrected brightest intensity in any of the first 25 sequencing cycles was less than 60% of the sum of brightest intensity and the next brightest. We find that this criterion provides reasonable discrimination between good and bad data [25]. Depending on the loading density, we typically kept between 50% and 70% of the raw reads. The final output was around 150 K reads on average per rat (range: 90–220 K). Operational taxonomic units (OTUs) were assigned using the 16S-metagenomics workflow (1.0.1) associated with the Base Space Hub (Illumina, 2013 version [26]). An Illumina-curated version of the GreenGenes taxonomic database, which implements the Ribosomal Database Project (RDP) Classifier was used to classify the OTUs [27]. The rarefaction curves (Supplementary Materials, Figure S1) show that the main OTUs are effectively detected. The Taxonomy Database (National Center for Biotechnology Information) was used for classification and nomenclature. Bacteria were classified based on the SCFA end product as previously described [28,29] (Supplementary Materials, Table S1). 

### 2.3. Statistical Analysis

Reads in each operational taxonomic unit (OTU) were normalized to total reads in each sample. Only taxa with a percentage of reads >0.001% were used for the analysis. The Shannon, Chao1, Simpsons and Pielou indices were calculated to analyze α-diversity using Past software (ver3.21, Oslo, Norway) [30]. Principal components analysis (PCA) [31] was also carried out with Past software [32]. Statistical taxonomic comparisons were performed using the linear discriminant analysis (LDA) effect size (LEfSe) using the Galaxy Hutlab online platform [33,34], considering alpha values of 0.05 for the factorial Kruskal-Wallis test among classes and for the pairwise Wilcoxon test between subclasses. Values > 2 or < −2 for the logarithmic LDA score were considered significant [35]. The Galaxy Hutlab web was also used to plot the cladogram. All other analysis and plots were carried out using Prism software (Prism version 7.04 for Windows, GraphPad Software, La Jolla California USA [36]). Normally distributed variables were compared using a two-tailed unpaired *t* test, otherwise data was analyzed using the Mann Whitney test. 

## 3. Results

### 3.1. Model of Vitamin D Deficit 

**Figure 1:**
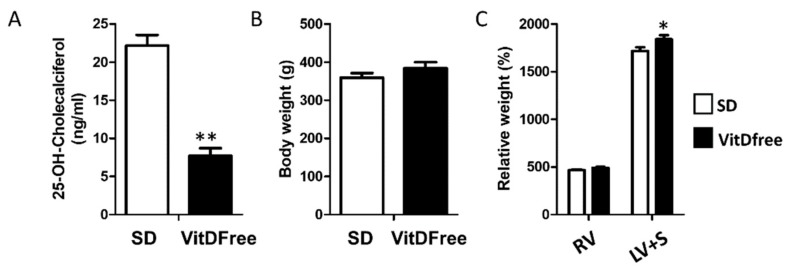
Vitamin D status, and body and heart weight. (**A**) Plasma 25OHVitD. (**B**) Body weight and (**C**) right ventricular (RV) and left ventricular + septum (LV + S) weights. Results are mean  ±  s.e.m. for the standard diet (SD, *n* = 10) and vitamin D-free diet group (*n* = 10). Results were compared by student’s t-test * *p* < 0.05 and ** *p* < 0.01 vs. SD.

The exposure to a vitamin D-free diet for 7 weeks induced a marked decrease in 25OHVitD plasma values from 22.2 ± 1.4 to 7.7 ± 1.0 ng/mL (Figure 1A). There was no change in body weight and a modest increase in the weight of the left ventricle plus septum but not in the right ventricle (Figure 1B,C).

### 3.2. Bacterial α- and β-Diversity

**Figure 2:**
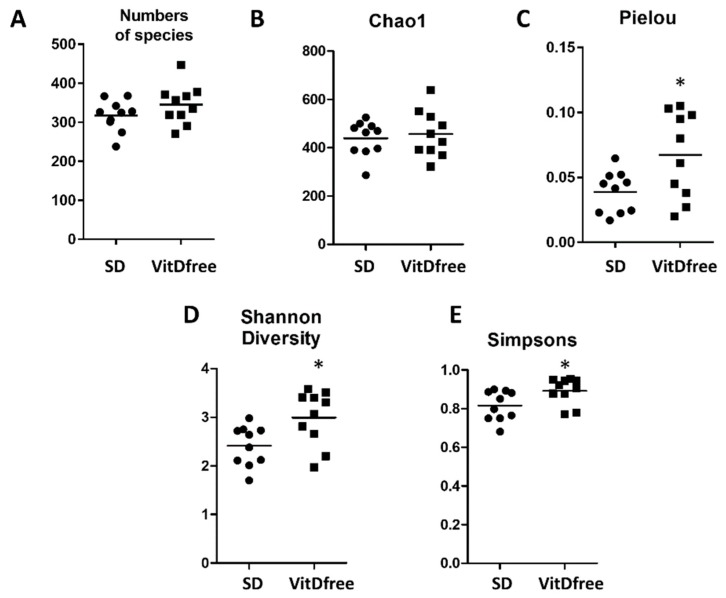
Effects of vitamin D-free diet on ecological parameters of the bacterial communities. Fecal samples were collected from standard diet (SD, *n* = 10) and vitamin D-free diet group (*n* = 10) rats. The microbial alpha diversity was analyzed using (**A**) numbers of species, (**B**) Chao richness, (**C**) Pielou, (**D**) Shannon and (**E**) Simpsons indices. Results are expressed as a scatterplot and means and were compared by a student’s t-test; * *p* < 0.05 vs. SD.

Between 250 and 450 species were identified in each sample. The number of species identified was similar in the rats under the standard diet and those with vitamin D-free diet (Figure 2A). The parameters indicating α-diversity such as the Pielou, Shannon and Simpson indices were significantly increased in vitamin D-free diet group but the Chao1 index was not significantly affected (Figure 2). 

**Figure 3:**
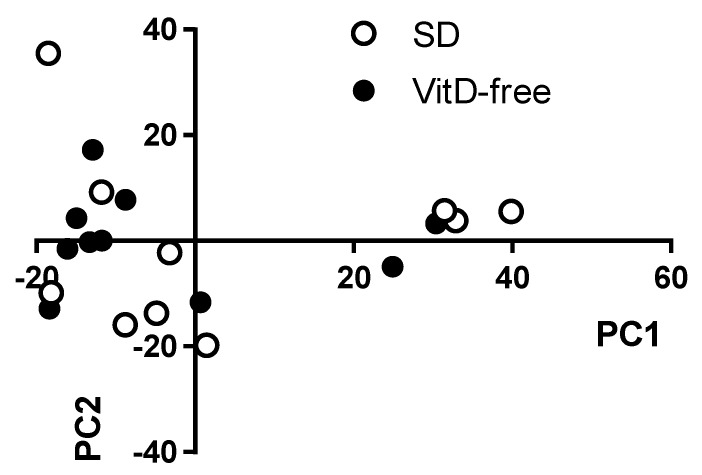
Effects of vitamin D-free diet on bacterial β-diversity. β-diversity was analyzed using the principal coordinate analysis (PCA) of the standard diet group using PAST3.0. The principal components PC1 and PC2 are plotted in a bidimensional figure. The white spheres represent rats fed a standard diet (SD, *n* = 10) and black spheres rats fed a vitamin D-free diet (*n* = 10).

A bidimensional principal component analysis (PCA) of the microbiome was performed in an unsupervised manner, which measures the diversity of microorganisms among samples, i.e., β-diversity. This analysis showed no clear clustering of the animals into the control and vitamin D-free diet groups (Figure 3). 

### 3.3. Taxa Composition

We performed an LDA effect size (LEfSe) analysis for the whole bacterial taxa identified in the feces to support high-dimensional class comparisons. The results of this analysis are shown in Supplementary Materials, Figure S2. Forty-one taxa were significantly increased in rats exposed to a VitD-free diet and five taxa were decreased. A cladogram summarizing the phylogenetic relationship between these changes is shown in Supplementary Materials, Figure S3. 

**Figure 4:**
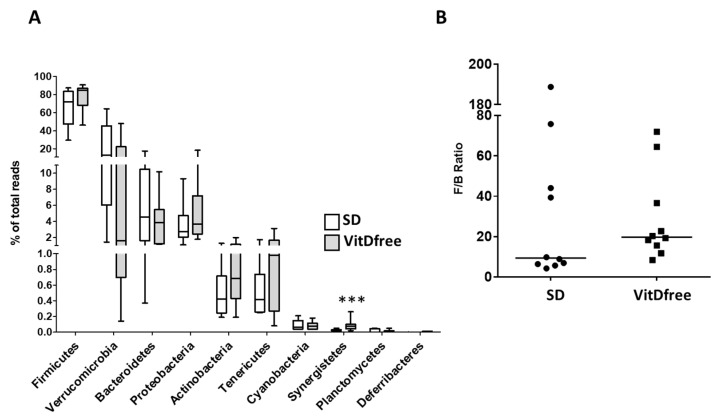
Effects of the vitamin D-free diet on the relative phylum abundance. The most abundant phyla for the standard diet (SD, *n* = 10) and vitamin D-free diet group (*n* = 10) were expressed as a percent of total reads and represented in (**A**) as a box and whiskers plot, *** *p* < 0.001 vs. SD, student’s t-test. (**B**) Firmicutes to Bacteroidetes ratio (F/B ratio), as a biomarker of gut dysbiosis, is represented as a scatterplot and median.

*Firmicutes* was the most abundant phylum in the rat feces, followed by *Verrucomicrobia*, *Bacteroidetes*, *Proteobacteria*, *Actinobacteria* and *Tenericutes*, in both groups (Figure 4A). Among these most abundant phyla there was no significant differences in their relative abundance when comparing animals with the standard diet and those with the vitamin D-free diet. We found a large variability in the *Verrucomicrobia* and to a lesser extent in the *Bacteroidetes* phylum. The ratio of *Firmicutes* to *Bacteroidetes* (F/B), which is a widely used measure of bacterial dysbiosis was highly variable (Figure 4B). There was an apparent trend for an increased F/B ratio in the Vit D-free group, the median increased from 9.3 to 19.7, but the difference was not statistically significant (*p* > 0.05, Mann Whitney test). Among the less abundant phyla, there was a highly significant increase in *Synergistetes* (Figure 4A).

**Figure 5:**
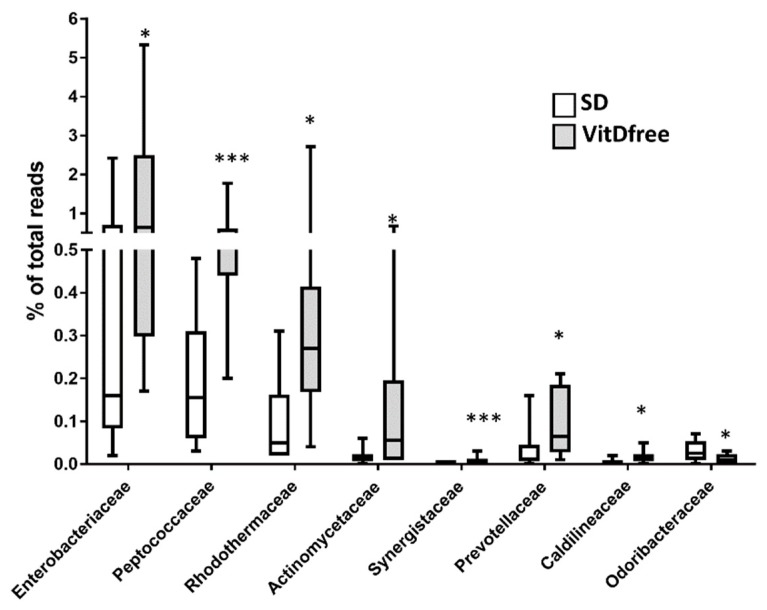
Effects of vitamin D-free diet on the relative family abundance. The most significant changes at the family level are shown as a percent of total reads and represented in a box and whiskers plot for the standard diet (SD, *n* = 10) and vitamin D-free diet group (*n* = 10). * *p* < 0.05 and *** *p* < 0.001 vs. SD, student’s t-test.

**Figure 6:**
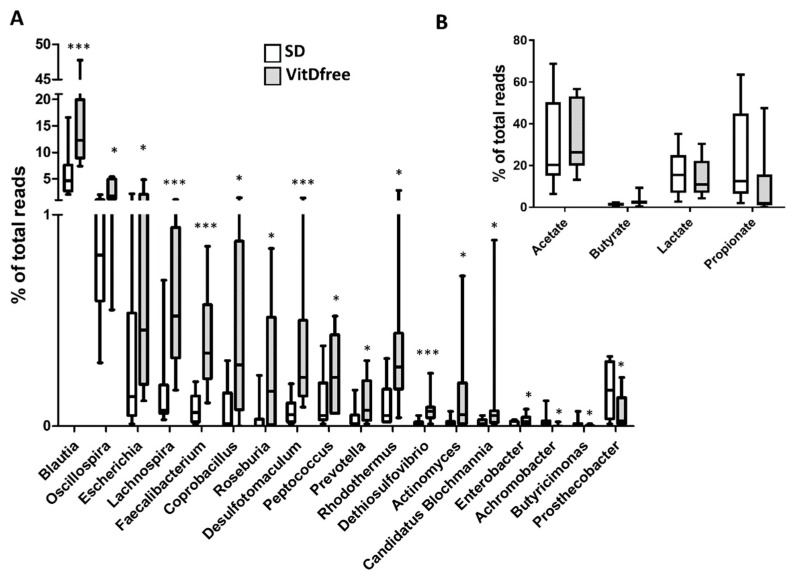
Effects of vitamin D-free diet on the relative genera abundance. (**A**) The most significant changes at the genus level are shown as a percent of total reads and represented in a box and whiskers plot for the standard diet (SD, *n* = 10) and vitamin D-free diet group (*n* = 10). (**B**). The relative proportions of acetate, butyrate, lactate and propionate producing bacteria in the gut microbiota. Sequence reads were classified according to the primary end product of the assigned bacterial genera. Genera were classified into more than one group correspondingly if they were defined as producers of multiple metabolites. Results were compared by student’s t-test; * *p* < 0.05 and *** *p* < 0.001 vs. SD.

The significant changes induced by vitamin D-free diet in bacterial families and genera are shown in Figure 5 and Figure 6A, respectively. Notably, there was an increase in the *Enterobacteriaceae* family with significant rises in its associated genera *Escherichia*, *Candidatus blochmannia and Enterobacter*, an increase in Prevotellaceae and its genus *Prevotella* and a decrease in the family Odoribacteraceae and its genus *Butyricimonas* in rats with vitamin D-free diet.

### 3.4. SCFA-Producing Bacteria 

We analyzed the changes in the relative abundance of SCFA-producing bacteria as an additional measure of gut dysbiosis (Figure 6B). Some specific acetate-producing genera such as *Blautia*, *Lachnospira*, *Actinomyces* and *Prevotella* were increased by vitamin D-free diet as mentioned above. Similarly, some butyrate-producing genera such as *Faecalibacterium* and *Roseburia* were relatively more abundant in rats with VitD-free diet. However, we found no significant changes in the sum of all bacterial reads for acetate-, butyrate-, lactate- or propionate-producing bacteria.

## 4. Discussion

The role of gut dysbiosis in the pathogenesis of many diseases, including diabetes mellitus, obesity, cancer, and psychiatric, respiratory and cardiovascular disorders is rapidly emerging. Similarly, vitamin D deficiency, which is the most common nutritional deficiency, has been related to a number of metabolic, cardiovascular, respiratory and psychiatric disorders. The primary end point of the present study was to analyze whether vitamin D deficiency induces gut dysbiosis. This might help to explain some of the health-related disorders associated with this hypovitaminosis. 

Determination of 25OHVitD plasma levels are the most reliable indicator of vitamin D storage. Values below 20 ng/mL are usually considered deficient [37,38] and are associated with secondary hyperparathyroidism, muscle weakness, osteomalacia or osteoporosis. We fed the rats for seven weeks with a VitD-free diet, leading to a severe vitamin D deficiency (mean plasma 25OHVitD <10 ng/mL). Animal studies of the microbiota, like the present one, can be performed in strictly controlled conditions of diet and environment. However, extrapolation of rodent models to human beings should be done with caution. For the first time we report vitamin D-free diet-induced changes in the microbiota in the rat, which is more representative of the human gut microbiota than mice [39]. For obvious reasons, a randomized controlled study inducing vitamin D deficiency in humans cannot be performed. To our knowledge, only a small controlled human study in patients with cystic fibrosis [40] has been carried out to study the opposite approach, i.e., to analyze the impact on the microbiota of restoring vitamin D levels in patients with vitamin D deficiency. The cutoff value of 25OHVitD used in this study to classify patients as deficient was 30 ng/mL and the post intervention mean values in the placebo arm was ≈ 25 ng/mL, which is often considered within the normal range, compared to the active VitD arm ≈ 46 ng/mL. Several non-controlled or observational human studies and some studies in mice have also been published [41].

Our results show that a VitD-free diet did not produce large changes in the gut microbiota characteristics of dysbiosis, i.e., it did not lower microbial α-diversity and richness, it did not increase the Firmicutes to Bacteroidetes ratio, and it did not change the relative abundance of SCFA-producing bacteria. Moreover, principal component analysis of the bacterial community could not discriminate VitD-free diet treated from control animals in an unsupervised manner. α-diversity represents both the richness and evenness of species bacterial diversity within each sample and it is usually analyzed using the Chao1, Pielou, Shannon and/or Simpsons indices and the number of species. They are commonly used as indirect indicators of a healthy bacterial community and associated with a better health status. However, paradoxically, these indices, with the exception of the Chao1 index, were modestly but significantly increased by the vitamin D-free diet. This reduced diversity may be associated with the antibacterial effects of vitamin D [23]. A similar trend was observed both in mice with colitis and in healthy mice exposed to a vitamin D-free diet compared to a standard diet [42]. Other studies analyzing the association between vitamin D and alpha diversity in human studies were not consistent [41].

Despite the lack of effect on the α- and β-diversity, vitamin D-free diet produced significant changes in the relative abundance of forty-nine taxa, with fourty-four increased and five decreased. The present study does not establish a causal relationship between the specific bacterial changes described herein and the potential health deleterious effect of vitamin D deficiency or the putative mechanisms involved. However, these changes resemble those found in other pathological conditions. Notably, with vitamin D-free diet we found significant rises in typical members of the oral and gut microbiota, which are often responsible for enteral, urinary and respiratory tract infections, such as Enterobacteriaceae and its genera *Escherichia* and *Enterobacter*. These genera belong to the class Gammaproteobacteria, which was significantly more abundant in the stool samples of vitamin D-insufficient subjects compared with vitamin D-sufficient subjects [42]. Other opportunistic bacteria, *Prevotella* and *Actinomyces*, commonly found in multiple types of infection, were also increased in vitamin D deficient rats. These data suggest that vitamin D deficiency increases the relative abundance of opportunistic pathogens which, in the context of intestinal barrier dysfunction, may favour pathogen bacterial translocation and systemic infection and inflammation. In fact, vitamin D preserves the intestinal epithelial barrier function [43]. Because the composition of the microbiota is essential for the intestinal barrier integrity, we speculate that the changes in the microbiota described herein may also be responsible for the vitamin D deficiency-induced alteration of the gut barrier. We also found a highly significant increase in bacteria from the phylum Synergistetes, which are considered as opportunistic pathogens involved in periodontitis [44], and its genus *Dethiosulfovibrio*, but its significance is unknown. Synergistetes and *Dethiosulfovibrionaceae* have also been reported in a rat model of acute myocardial infarction [45]. 

The family Odoribacteraceae and its genera *Odoribacter* and *Butyricimonas* have been reported to be depleted in several pathological conditions and animal models of disease such as overweight and obese pregnant women with high blood pressure, in sedentary mice, liver injury and pulmonary hypertension [13,46,47]. We found that this family and *Butyricimonas*, were decreased in vitamin D deficient rats. Likewise, this family was increased in response to vitamin D supplementation in vitamin D-insufficient subjects with cystic fibrosis [40]. Interestingly, *Odoribacter* was also depleted in vitamin D receptor knockout mice suggesting that this change is a VDR-mediated effect [48]. The potential protective effect of these genera is unclear. However, the abundance of *Butyricimonas*, which was decreased after high fat diet and reversed by statins or fecal transplantation, was correlated with the inflammatory cytokines IL-1β and TGFβ1 in the ileum, suggesting an anti-inflammatory effect [49].

## 5. Conclusions

In conclusion, vitamin D deficiency does not induce gut dysbiosis in the rat but produced specific changes in bacterial taxa, which may play a pathophysiological role in the immunologic dysregulation associated with this hypovitaminosis.

## Supplementary Materials

The following are available online at https://www.mdpi.com/2072-6643/11/11/2564/s1.
