# Supplementary material for: Impact of Vitamin D Deficit on the Rat Gut Microbiome
Source: Nutrients. 2019 Oct 24;11(11):2564. doi: 10.3390/nu11112564 (PMC6893507; doi:10.3390/nu11112564)
Supplement: Supplementary file 1 [file nutrients-11-02564-s001.pdf]

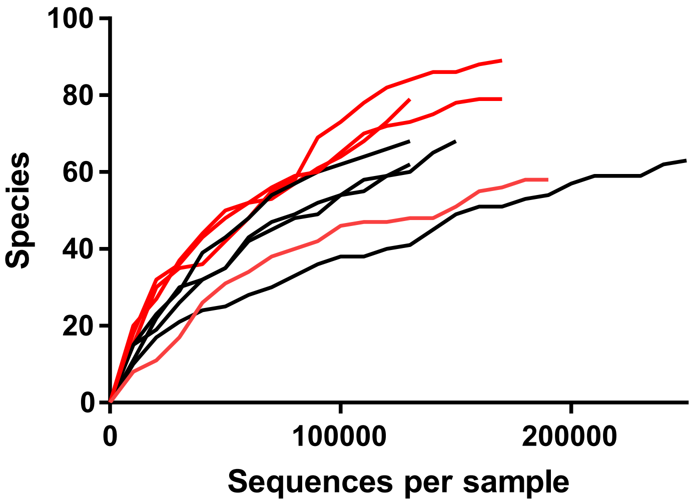

Supplemental Figure 1. Rarefaction curves. The rarefaction curves were analyzed in the first set of experiments (n = 4 for controls in black and n = 4 for vitamin D-free diet in red). Above 50.000 reads there is not linear increase in the number of species detected. Therefore, the number of reads was kept above 100.000 (156.000 ± 27.000, 2x300 pass-filtered paired-end) for the whole project.

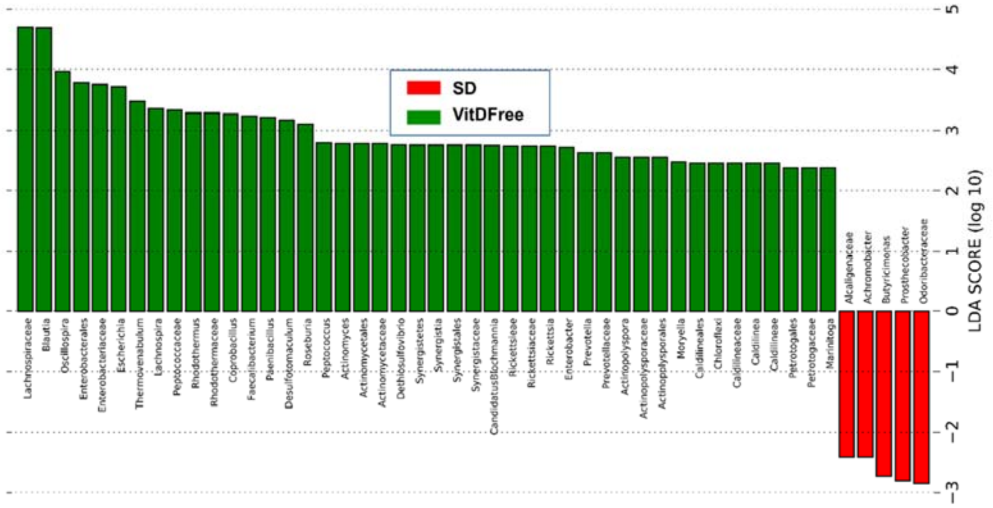

Supplemental Figure 2. Most enriched and depleted phyla, classes, orders, families and genera based on a linear discriminant analysis (LDA). The enriched bacterial taxa in the standard and in the vitamin D free diet group are shown in red and green bars, respectively. The LDA score was calculated using LefSE and the significant changes shown were analyzed based on Kruskal-Wallis test. The alpha value and the threshold LDA score were set to 0.05 and 2.0, respectively.

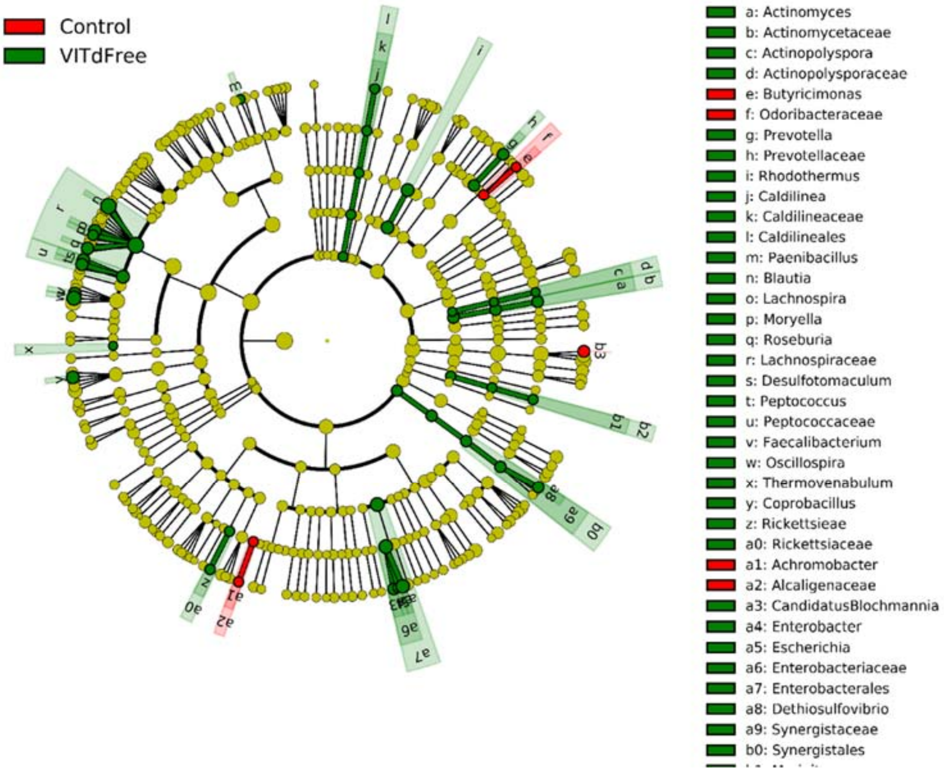

Supplemental Figure 3. Cladogram illustrating phylogenetic relationship between significantly changed taxa. The center dot represents the kingdom Bacteria, the first circle represents Phylum, then Class, Order, Family, and Genus levels, respectively. To construct the cladogram data was analyzed based on a Kruskal-Wallis test. The enriched taxa in the standard and in the vitamin D free diet group are shown in red and green, respectively. The alpha value and the threshold LDA score were set to 0.05 and 2.0, respectively.

Supplemental Table 1. Short Chain fatty acid producing bacteria.

Major genera identified in our 16S deep sequencing data were classified according to their primary fermentation products as acetate, butyrate or lactate using Bergey's Manual of Systematic Bacteriology. For several genera, propionate was a co-product of fermentation, not their major product. Those genera that produce succinate, ethanol and H+ as their primary fermentative by-products were not included in the analysis shown in Figure 6. The genera with unknown or ambiguous fermentative products were excluded. Major genera were classified according to the dominant fermentation end-product(s) as shown below. Abbreviations: A=acetate, B=butyrate, L=lactate, P=propionate.

Acetanaerobacterium	A
Acetitomaculum	A
Acidaminococcus	A, B
Acidaminococcus	B
Acidaminococcus	A, B
Actinomyces	A
Akkermansia	A,P
Anaerofilum	A
Anaerofustis	A,B
Anaerofustis	A,B
Anaeroglobus	A,B
Anaeroglobus	B
Anaeroplasma	A
Anaerosporobacter	A
Anaerostipes	B
Anaerotruncus	A,B
Anaerotruncus	A,B
Asteroleplasma	A
Atopobium	A
Bacteroides	A
Bifidobacterium	L
Bilophila	A
Blautia	A
Butyricicoccus	B
Butyricimonas	B

Butyrivibrio	B
Cellulosilyticum	A
Christensenella	A
Cloacibacillus	A,B
Cloacibacillus	B
Coprococcus	B
Corynebacterium	L
Corynebacterium_1	L
Corynebacterium_4	L
Desulfobulbus	A
Dialister	A
Dorea	A
Dysgonomonas	P,L
Enterococcus	L
Eubacterium	B
Faecalibacterium	B
Fusobacterium	B
Gemella	A
Holdemania	A
Lachnospira	A
Lactobacillus	L
Lactococcus	L
Leuconostoc	A
Lutispora	A
Marvinbryantia	A
Moryella	A,B,L
Moryella	B
Moryella	A,B,L
Oribacterium	A
Parabacteroides	A
Paraprevotella	A

Parvimonas	A
Pediococcus	L
Prevotella	A
Pseudobutyrivibrio	B
Pseudoflavonifractor	A
Pseudoramibacter	A,B
Pseudoramibacter	A,B
Pyramidobacter	A
Robinsoniella	A
Roseburia	B
Shuttleworthia	A,B
Shuttleworthia	A,B
Staphylococcus	L
Streptococcus	L
Subdoligranulum	B
Tannerella	A,B
Turicibacter	L
Victivallis	A
Weissella	L
Xylanibacter	A
